# Editorial: Investigating the elements of plant defense mechanisms within plant immune responses against pathogens, volume II

**DOI:** 10.3389/fpls.2026.1846947

**Published:** 2026-05-06

**Authors:** Katarzyna Otulak-Kozieł, Edmund Kozieł, Józef Julian Bujarski

**Affiliations:** 1 Department of Botany and Plant Physiology, Institute of Biology, Warsaw University of Life Sciences-SGGW, Warsaw, Poland; 2 Department of Biological Sciences, Northern Illinois University, DeKalb, IL, United States

**Keywords:** abiotic stress, biotechnology, climate change, physiology, plant structure, plant-disease, plant-pathogen interactions, signaling

The inherent connection of plants with their growth habitats where they “conceive” offspring and release its new generation imposes a constant pressure to interact with various environmental factors, including those that are pathogenic or pest-related (Jian et al., 2024). Therefore, plants need to develop or enhance defense mechanisms to sustain their existence. The coordinated multi-layered defense system includes early physiological response, rapid activation of defense enzymes, substantial accumulation of reactive oxygen species (ROS), and swift and robust activation of transcriptional reprogramming to develop resistance (Liu et al.; Haghpanah et al., 2025) or control the interacting microorganisms during symbiotic interactions. Various types of pathogens, including viruses (Shang et al.; Zhao et al.; Choi; Kan and Citovsky), bacteria (Liu; Yue et al.), fungi (Abdelghany et al.; Talmo and Ranjan; Maravilha et al.; Wu et al.), and pest insects (Xie et al.), as well as symbiotic microorganisms (Aseel et al.) significantly influence plant growth and crop productivity (Lei et al.; Sun et al.) by inducing direct/indirect changes in plant metabolism and structure. This situation highlights the need to continually enhance our understanding of the complex interaction network of symbiotic/pathogenic organisms with plants, particularly because plants sustain life on Earth and serve as a primary food source for the world’s population (Otulak-Kozieł et al., 2024).

This Research Topic was aimed to elucidate the implications of understanding how pathogens change adaptive mechanisms to infect plants or how plants develop diverse resistance; furthermore, it explores how antimicrobial or chemical treatments can be utilized to enhance plant resilience.

Maravilha et al. showed that *Lathyrus sativus* utilizes complex molecular interactions and defense mechanisms against *Erysiphe pisi*, underscoring the genetic diversity in pathogen response across accessions with contrasting resistance levels. The authors identified novel targets such as NOD-like receptors (NLRs) and effectors, antifungal proteins, and cell wall reinforcement-related genes that participate in the complex *L. sativus*-*E. pisi* interaction; this finding could support future breeding programs aimed at enhancing the resistance of *L. sativus* and related species to *E. pisi*. Liu revealed that the FLiS protein triggered a hypersensitive response (HR) in tobacco and elicited an immune response in cotton. The FLiS protein was hypothesized to potentiate the immune response against *Verticillium dahliae* infection by modulating the jasmonic acid (JA) and salicylic acid (SA) signaling pathways. The author’s results suggest that FLiS has a promising potential as a bioregulator for augmenting the resistance of upland cotton to *V. dahliae*. Yue et al. analyzed the synergistic effect between the *Arabidopsis* U-box protein PUB13 and the copine-like protein BON1 in the regulation of pathogen-associated molecular pattern-triggered immunity (PTI). The authors emphasized that the activity of E3 ubiquitin ligase is required for PUB13 to regulate biological functions and the synergistic interaction of BON1 with PUB13 regulates not only immunity but also plant growth and flowering in *Arabidopsis* plants. By integrating agronomic, anatomical, biochemical, and molecular traits, Abdelghany et al. highlighted the complexity of resistance of maize to LWD (late wilt disease) caused by *Magnaporthiopsis maydis* and underscored the importance of selecting genotypes that combine high yield potential with disease resistance mechanisms, offering important insights for breeding climate-resilient maize and advancing sustainable disease strategies. In a recent study, Liu et al. integrated transcriptomic and physiological analyses to elucidate the defense mechanisms of *Bletilla striata* (*Orchidaceae*) against leaf rust disease caused by *Coleosporium bletiae*. The authors indicated that significantly upregulated differentially expressed genes (DEGs) associated with antioxidant defense systems, secondary metabolite biosynthesis, and JA, SA, and brassinosteroid (BR) signaling pathways, in contrast to downregulated DEGs involved in cell wall remodeling, and calcium-mediated signaling are the main components that regulate molecular and biochemical responses, which effectively restricted pathogen colonization and dissemination. Moreover, Wu et al. reported on Cercospora leaf spot (CLS), which is a major threat to sugar beet (*Beta vulgaris L.*) and is caused by *Cercospora beticola*. Transcriptome profiling, subcellular localization, and physiological observations shed light on the potential roles of abscisic acid (ABA) receptors in plant defense responses against fungal pathogens, providing a deeper understanding of the ABA signaling pathway and its interaction with biotic stresses in sugar beet. The review article by Talmo and Ranjan provides an overview of defense strategies against Sclerotinia stem rot (SSR) in soybean and canola as well as head rot (SHR), mid-stalk rot (MSR), and basal stalk rot (BSR) in sunflower. By identifying commonalities and differences among distantly related hosts, the authors enhanced our understanding of key plant defense strategies against *Sclerotinia sclerotiorum*, simultaneously highlighting areas requiring future research. Another review by Lei et al. underlined the roles of sugar transporters as a novel strategy for enhancing crop health and productivity. The authors systematically categorized plant sugar transporters and emphasized their functional significance in mediating plant interactions with pathogenic and beneficial microorganisms.

The research groups Shang et al., Zhao et al., Choi, and Aseel et al. revealed their recent findings related to plant–virus interactions. Choi focused on the incidence rates of viral diseases caused by East Asian Passiflora virus (EAPV), Euphorbia leaf curl virus (EuLCV), Cucumber mosaic virus (CMV), and Papaya leaf curl Guandong virus (PaLCuGdV); the author found that the incidence of these diseases increases with the increase in cultivation years. The article provides foundational data to increase the stability of *Passiflora edulis* cultivation against plant viral infections and prevent the spread of viral diseases. Kan and Citovsky targeted two viral proteins: the movement protein and the coat protein, which are directly involved in the local and systemic spread of tobamoviruses, with respect to their phylogeny, activities during viral movement, and interactions with the host determinants of the movement process. Tospoviridae tomato spotted wilt virus (TSWV) infection with cytosine-5 methylation (m5C) epitranscriptomic map in *Solanum lycopersicum* and its role in plant immunity were the topics of research conducted by Zhao et al. The authors revealed that key RNA methyltransferase gene *(SlTRM4B)*-mediated m5C RNA methylation fine-tunes post-transcriptional regulation to reprogram the transcriptome for disease resistance in tomato. The data reported by Shang et al. provide new observations related to balancing growth and plant defense under fluctuating environmental conditions and its critical role in plant resilience. Shang et al. identified antagonistic miRNA hubs: *miR397a* promotes susceptibility to Soybean mosaic virus through laccase repression, whereas *miR399j* enhances resistance to this virus by remapping phosphorus allocation through *GmPHT1–4* targeting, reducing root: shoot ratios, and directing more phosphorus content to leaves to enhance defense metabolism. The authors also highlighted that both pathways are inhibited under shade, forcing a light-gated resource hierarchy that sacrifices immunity for metabolic maintenance. Additionally, Aseel et al. presented studies where arbuscular mycorrhizal fungi (AMF) synergize with *Trichoderma* spp. to improve plant nutrition and stimulate systemic plant defenses in response to viral pathogen challenges. Moreover, the dual application of AMF and *Trichoderma* spp. significantly suppressed viral symptoms and decreased viral accumulation levels and disease severity. Furthermore, both single and dual treatments substantially enhanced the activity of antioxidant enzymes, chlorophyll concentration, and macronutrient levels in tomato tissues. These new findings provided by the authors indicate that AMF colonization and *T. viride* foliar spraying enhance tomato resistance to potato virus Y by activating defense systems, highlighting their crucial role in inhibiting plant viruses.

This Research Topic reveals new insights, current challenges, and latest discoveries, with prospects in the field of plant defense mechanisms related to plant immune response against various pathogens and pests. Despite the extensive studies reviewed here, further research is required to provide scientific support to improve disease prevention and control in plants. We would like to thank all authors and reviewers for their contributions, and we hope this Research Topic stimulates further interdisciplinary research for understanding the broad spectrum and underlying elements of defense mechanisms in plant–pathogen interactions as an essential approach to improve plant resistance to pathogens.
